# Of goals and habits: age-related and individual differences in goal-directed decision-making

**DOI:** 10.3389/fnins.2013.00253

**Published:** 2013-12-24

**Authors:** Ben Eppinger, Maik Walter, Hauke R. Heekeren, Shu-Chen Li

**Affiliations:** ^1^Chair of Lifespan Developmental Neuroscience, Department of Psychology, TU DresdenDresden, Germany; ^2^Center for Lifespan Psychology, Max Planck Institute for Human DevelopmentBerlin, Germany; ^3^Department of Education and Psychology, Freie Universität BerlinBerlin, Germany

**Keywords:** aging, decision-making and learning, dopamine, goal-directed, habitual

## Abstract

In this study we investigated age-related and individual differences in habitual (model-free) and goal-directed (model-based) decision-making. Specifically, we were interested in three questions. First, does age affect the balance between model-based and model-free decision mechanisms? Second, are these age-related changes due to age differences in working memory (WM) capacity? Third, can model-based behavior be affected by manipulating the distinctiveness of the reward value of choice options? To answer these questions we used a two-stage Markov decision task in in combination with computational modeling to dissociate model-based and model-free decision mechanisms. To affect model-based behavior in this task we manipulated the distinctiveness of reward probabilities of choice options. The results show age-related deficits in model-based decision-making, which are particularly pronounced if unexpected reward indicates the need for a shift in decision strategy. In this situation younger adults explore the task structure, whereas older adults show perseverative behavior. Consistent with previous findings, these results indicate that older adults have deficits in the representation and updating of expected reward value. We also observed substantial individual differences in model-based behavior. In younger adults high WM capacity is associated with greater model-based behavior and this effect is further elevated when reward probabilities are more distinct. However, in older adults we found no effect of WM capacity. Moreover, age differences in model-based behavior remained statistically significant, even after controlling for WM capacity. Thus, factors other than decline in WM, such as deficits in the in the integration of expected reward value into strategic decisions may contribute to the observed impairments in model-based behavior in older adults.

## Introduction

Many simple everyday decision-making tasks, such as which cereals to take for breakfast or which subway to take to work in the morning, can be solved via habitual decision mechanisms. However, in more complex decision scenarios, such as how to spend annual bonus or how to plan retirement savings, it may be adaptive to anticipate the consequences of future decisions and to choose the options that are likely to yield higher long-term benefits. In the current study we examined age and individual differences in the interplay between habitual and goal-directed decision-making. We had three specific research questions in mind: first, does aging affect the balance between habitual and goal-directed decision mechanisms? Second, are age differences in the interplay of these decision mechanisms related to age differences in working memory (WM) capacity? Third, can model-based choice behavior be affected by manipulating the distinctiveness of the reward value of different choice options? To address these questions we adapted a two-state Markov decision task (Daw et al., 2011; Wunderlich et al., 2012) in combination with computational reinforcement learning (RL) modeling.

### Model-free and model-based decision-making

The dissociation between habitual and goal-directed mechanisms is at the core of many current theories of learning and decision-making (Daw et al., 2005; Balleine and O'Doherty, 2010; Kahneman, 2011). Habitual or model-free learning refers to the acquisition of behavior based on associations between actions and effects: actions that are followed by reward are more likely to reoccur (Thorndike, 1911). Model-free learning is a robust and computationally efficient mechanism. However, it can come at the cost of being inflexible, especially in dynamically changing environments, which constrain the adaptive value of habitual responses (Doll et al., 2012). Computational accounts suggest that habitual learning is driven by the discrepancy between the current reward and the expected plus the (discounted) sum of all future rewards (i.e., the prediction error signal) (Sutton and Barto, 1998; Niv and Schoenbaum, 2008). Results from electrophysiological studies in animals and neuroimaging work in humans show that these reward predictions errors seem to be coded in phasic changes of dopaminergic activity in the midbrain and ventral striatum (Schultz et al., 1997; Montague et al., 2004; D'Ardenne et al., 2008; Niv et al., 2012).

In comparison, goal-directed or model-based decision-making reflects choices that are guided by internal goal representations or “cognitive maps” (Tolman, 1948; Miller and Cohen, 2001). These representations involve knowledge of the structure of the environment that can be used to make adaptive and foresighted decisions (Doll et al., 2012). One way of thinking about these representations is in terms of a decision space that represents the consequences of actions with respect to sequential transitions in the environment and possible future rewards. The advantage of model-based decision mechanisms is that they allow individuals to flexibly adjust behavior to changes in the environment. One downside of model-based decision-making is that it is computationally more expansive and effortful than the relatively more automatic habitual mechanisms. Recent neuroimaging work has started to investigate the neural mechanisms underlying model-based decision-making (Gläscher et al., 2010; Daw et al., 2011). Whereas results from Daw et al. (2011) suggest that model-based and model-free decisions may implicate overlapping neural systems, involving the ventral striatum and ventromedial prefrontal cortex (vmPFC), findings from Gläscher et al. (2010) show that the learning of new task structures may involve cortical areas such the lateral PFC and parietal cortex.

### Relations between working memory capacity and model-based decision-making

Support for the idea that model-based decision-making relies on higher-order cognitive control mechanisms comes from a recent study that combined a two-state Markov decisions task with a concurrent WM paradigm (Otto et al., 2013). This study showed that high WM load resulted in a reduced degree of model-based behavior, suggesting that goal-directed decisions rely on WM functions and the associated neural systems. Similar results were obtained by Worthy and Maddox (2012). Using a dynamic decision-making task these authors showed that WM load seems to shift behavior from a heuristics-based win-stay lose-shift (WSLS) strategy toward a model-free (reinforcement-based) strategy (Worthy and Maddox, 2012). Taken together, evidence from these studies suggests that model-based decision-making may partially rely on WM function and that increasing WM demands may lead to a shift from model-based to model-free decision-making. Another implication from these findings is that model-based decision-making abilities can be understood as a limited cognitive resource. However, what remains unclear from these studies is the degree to which age and individual differences in WM capacity may be associated with differences in model-based behavior (Otto et al., 2013).

### Age differences in learning and decision-making

Results from recent studies on age differences in learning and decision-making suggest that older adults are impaired in learning from uncertain and ambiguous reward. This does not seem to be the case in situations in which reward information is fully predictable (deterministic). Electrophysiological results indicate that learning impairments are associated with deficits in error detection as well as less differentiated reward representations (Eppinger et al., 2008; Eppinger and Kray, 2011; Pietschmann et al., 2011; Hämmerer and Eppinger, 2012). Moreover, results from recent fMRI studies show that age-related impairments in RL are associated with a reduced correlation between reward prediction errors and ventral striatal activity in older than younger adults (Chowdury et al., 2013; Eppinger et al., 2013). In line with the idea of age-related changes in striatal prediction error signaling, Samanez-Larkin et al. (2010) found that suboptimal financial decision-making in older adults is associated with increased temporal variability of the ventral striatal BOLD signal. Taken together, these results are consistent with several theoretical proposals, suggesting that age-related impairments in reward-based learning might result from reduced dopaminergic projections from the midbrain to the ventral striatum and vmPFC (Nieuwenhuis et al., 2002; Frank and Kong, 2008; Hämmerer and Eppinger, 2012). However, it should be noted that there is also evidence indicating that age-related deficits in learning are, at least partially, mediated by decreased white matter integrity in fronto-striatal pathways (Samanez-Larkin et al., 2012).

Only a few studies so far have focused on age-related differences in more complex learning and decision-making (Mata et al., 2010; Worthy et al., 2011; Worthy and Maddox, 2012). In a recent study Worthy and Maddox (2012) used a dynamic decision-making task in which reward depended on the choice history. Results showed that older adults performed better on this task than younger adults. Using computational approaches the authors showed that this effect was due to the fact that older adults relied more on decision heuristics such as a win-stay lose-shift, whereas younger adults relied on RL. Findings by Mata et al. (2010) show that older adults perform poorer in a probabilistic inference task than younger adults if the decision environment favors the use of a cognitively demanding strategy. This is consistent with the idea that strategic, planning-related cognitive processes are a constrained resource, especially in older adults. Taken together, these results point to the view that age-related impairments in decision-making may depend on the complexity of the decision environment (Mata et al., 2010). Older adults may do well or even better than younger adults in tasks that favor the use of decision strategies with shorter temporal horizons, such as WSLS, whereas older adults may be impaired in decision-making if they have to use strategic, model-based processes.

**Figure 1:**
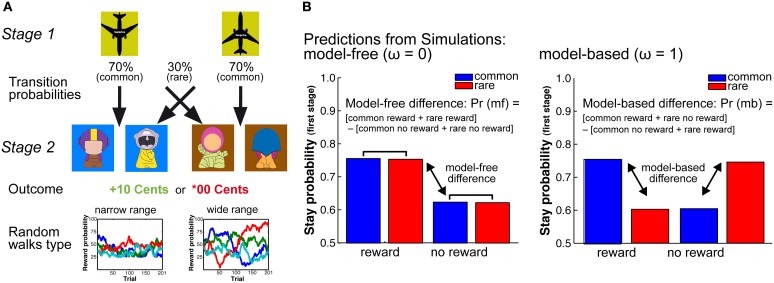
**(A)** Schematic Figure of the 2-stage Markov decision task. In this task participants have to constantly update reward predictions on the second stage (model-free decision-making) and use these reward predictions to make goal-directed decisions on the first stage of the task. To support model-free learning in the two age groups we manipulated the random walks that determine the probability of getting a reward at the second stage. We applied two different probability ranges, one with a narrow range of reward probabilities (25–75% reward probability) and one with a wide range of reward probabilities (0–100% reward probability). **(B)** Model predictions. Left panel: Simulations show that model-free decision-making is reflected in a main effect of reward. That is, stay behavior on the first choice depends on whether behavior on the previous trial was rewarded or not. Model-free behavior is independent of the transition probability structure. Right panel: Model-based behavior is reflected in an interaction between transition on the previous trial and reward on the previous trial. That is, model-based behavior takes the model-free information as well as knowledge of the transition structure into account.

Although the results of the previous studies may point to an age-related shift in the balance between model-based and model-free decision processes, the tasks applied in these studies do not allow to formally dissociate between these decision mechanisms. To address this question and to examined age and individual differences in model-free and model-based decision processes, we adapted a two-stage Markov decision task (cf. Daw et al., 2011) that allowed us to separate the contributions of these decisions mechanisms to choice behavior (see Figure 1A).

Specifically, we were interested in three major research questions (a) Does aging affect the balance between model-based and model-free decision-making mechanisms? (b) Are age-related changes in decision mechanisms related to age differences in WM capacity?; and (c) Can model-based behavior in older adults be supported by enhancing the distinctiveness of the reward value of the different choice options?

The two-stage Markov decision task consists of two decision stages in each trial (see Figure 1A). The first decision stage involves two choice options that are associated with different transition probabilities to the second-stage choice options that are then either rewarded or not rewarded. In this task, participants have to constantly update reward predictions at the second stage (model-free decision-making) and use this information prospectively to make goal-directed (model-based) decisions at the first decision stage on the next trial (see Figure 1A). To manipulate the demands on the representation and updating of reward value we varied the distinctiveness of the reward probabilities at the second stage (see Figure 1A). This was done by increasing the range of the reward probabilities of the choice options at the second stage on each trial. The larger the range of the reward probabilities of the four potential choice options, the easier it should be to differentiate and represent the reward histories associated with these options (cf. Eppinger et al., 2011). More differentiable reward probabilities on the second stage should support the ability to make deliberate, goal-directed decisions on the first stage and may hence be less demanding in terms of the representation of the stage transition structure of the task.

Given previous findings that point to age-related behavioral deficits in complex decision tasks (Mata et al., 2010) we expected older adults to be impaired in model-based decision-making compared to younger adults. Furthermore, given evidence for age-related impairments in learning from probabilistic outcomes (Eppinger et al., 2008; Hämmerer et al., 2011; Pietschmann et al., 2011), we predicted that older adults should benefit from more distinctive reward probabilities at the second stage of the task. That is, we should find enhanced model-based decision-making in the wide compared to the narrow probability range condition. To investigate the association between individual differences in WM and individual differences in model-based decision-making we also acquired a WM capacity using the operation span task (Turner and Engle, 1989; Unsworth et al., 2005). Given results suggesting that WM capacity is critical for model-based behavior we expected that higher WM capacity should be associated with model-based choice patterns (Otto et al., 2013). To the degree that this is the case, age-related deficits in model-based decision-making may be mediated by age-related decline in WM capacity (Salthouse et al., 1989; Salthouse, 1994).

## Material and methods

### Participants

Sixty younger and sixty two older adults took part in the study. Two older adults had to be excluded because they were unable to perform the experimental tasks. Two younger adults were excluded because they did not return for the second testing session. Two further younger adults needed to be excluded, one due to technical problems during data acquisition and the other due to chance level performance in the WM task. Thus, the effective sample consisted of 56 younger adults (mean age: 24, age range 20–30 years, 27 females) and 60 older adults (mean age: 69, age range: 56–78 years, 27 females). Participants gave written informed consent. The Institutional Review Board of the Max-Planck Institute for Human Development approved the study. Participants completed a biographical and a personality questionnaire (Carver and White, 1994) as well as several psychometric tests: (1) Identical pictures test (Ekstrom et al., 1976); (2) Raven's Progressive matrices (Raven et al., 1998); (3) Spot-the-Word test (Baddeley et al., 1992). As shown in Table 1 older adults had lower scores on the Identical Pictures test and Raven's matrices than younger adults (*p*'s < 0.001, η^2^ > 0.46). In contrast, older adults obtained higher scores than younger adults on the Spot-the-Word test (*p* < 0.001, η^2^ = 0.24). Consistent with previous findings from larger population-based samples (e.g., Li et al., 2004), these results suggest age-related reductions in fluid intelligence and age-related improvements in crystallized intelligence. We did not find significant age differences behavioral inhibition or approach (BIS/BAS) scores (*p*'s > 0.48) (Carver and White, 1994).

**Table 1:** **Psychometric variables displayed separately for the two age groups and the two working memory performance groups**.

**Age group**	**Younger adults**		**Performance effect**	**Older adults**		**Performance effect**	**Age effect**
**WM performance groups**	**Low (mean, *SE*)**	**High (mean, *SE*)**	***p*-value, effect size**	**Low (mean, *SE*)**	**High (mean, *SE*)**	***p*-value, effect size**	***p*-value, effect size**
Age	25.2 (0.6)	23.8 (0.6)	*p* = 0.08, η^2^ = 0.06	69.1 (1.0)	68.4 (0.9)	*p* = 0.60, η^2^ = 0.00	*p* < 0.001, η^2^ = 0.97
Raven	11.3 (0.5)	12.9 (0.5)	*p* = 0.03, η^2^ = 0.08	5.7 (0.5)	7.4 (0.7)	*p* = 0.05, η^2^ = 0.07	*p* < 0.001, η^2^ = 0.46
Ospan partial score	20.7 (1.5)	49.1 (1.9)	*p* < 0.001, η^2^ = 0.72	9.2 (1.1)	34.6 (2.1)	*p* < 0.001, η^2^ = 0.67	*p* < 0.001, η^2^ = 0.14
Ospan total score	45.4 (2.0)	63.6 (0.9)	*p* < 0.001, η^2^ = 0.56	26.3 (2.2)	53.3 (1.4)	*p* < 0.001, η^2^ = 0.66	*p* < 0.001, η^2^ = 0.20
Processing speed	29.0 (1.0)	33.8 (0.7)	*p* < 0.001, η^2^ = 0.25	22.03 (0.6)	21.5 (0.7)	*p* = 0.55, η^2^ = 0.00	*p* < 0.001, η^2^ = 0.57
Spot-a-word	19.5 (1.1)	20.4 (1.3)	*p* = 0.58, η^2^ = 0.00	25.4 (1.0)	26.7 (0.9)	*p* = 0.33, η^2^ = 0.02	*p* < 0.001, η^2^ = 0.24
BIS	19.5 (0.7)	18.82 (0.8)	*p* = 0.50, η^2^ = 0.00	19.86 (0.6)	17.93 (0.6)	*p* = 0.03, η^2^ = 0.08	*p* = 0.69, η^2^ = 0.00
BAS	13.5 (0.3)	13.6 (0.3)	*p* = 0.82, η^2^ = 0.00	13.5 (0.4)	13.0 (0.3)	*p* = 0.29, η^2^ = 0.02	*p* = 0.48, η^2^ = 0.00

### Median split of groups based on working memory capacity

To examine the associations between individual differences in WM capacity on model-based and model-free decision-making we performed a median split for the operation span total score separately for the two age groups (Unsworth et al., 2005). High and low capacity groups did not differ significantly with respect to mean age (younger adults: *p* = 0.08, older adults: *p* = 0.60, see Table 1). However, as expected, given the well-documented positive association between WM capacity and fluid intelligence (Duncan et al., 2012), we found significantly higher Raven scores for high than low WM capacity groups in both age groups (*p*'s < 0.05; η^2^'s > 0.07). Significant differences between high and low capacity in processing speed were observed for younger (*p* < 0.001; η^2^ > 0.25), but not for older adults (*p* = 55). High and low WM capacity groups did not differ with respect to semantic knowledge in either age group (*p*'s > 0.33) (see Table 1).

### Stimuli

Stimuli on the first stage were two airplanes that either pointed to the top or the bottom of the screen, indicating the two different choice options. Stimuli on the second stage were 8 colored figures (“GoGos”) that we generated using a freeware on the gogos-crazybones.com website and processed for presentation purposes in Photoshop (see Figure 1A). Background colors of the second stage stimuli were either blue or brown. Feedback stimuli either indicated a monetary gain of 10 Euro Cents, displayed in green or a neutral outcome of 00 Euro Cents, displayed in red (see Figure 1A). Stimuli were presented on a 19-inch computer screen using the program EPrime 2.0 software (PST Inc., Pittsburgh, PA).

### Task

The task involves two decision stages. At the first stage participants had to make a decision between two choice options (the two airplanes), which occurred randomly either on the left or right side of the screen. This decision determined the transition to the next (second) stage (see Figure 1A). We refer to more likely (70%) transitions as common transitions and less likely (30%) transitions as rare transitions. Participant had to indicate their choice within 2 s of stimulus presentation using the “f” or “j” key on a standard computer keyboard. If no response occurred within 2 s the trial was aborted and a new trial started. At the second stage participants had to make another decision between two choice options (the GoGos), which were displayed randomly either on the left or the right side of the screen (see Figure 1A). This decision had to be made within 2 s of stimulus presentation using the same keys as in the first decision (“f” and “j”). If no response occurred within 2 s, three white question marks appeared on the screen for 1 s and the trial was aborted. Choices were either rewarded (+10 Cents) or not rewarded (^*^00 Cents). The probabilities of getting a reward were determined by Gaussian random walks (see Figure 1A). The feedback stimuli were displayed for 1 s. Before and after the feedback stimulus a fixation cross was displayed for 500 ms. Reward probabilities were determined by a slowly drifting random walk. At each trial we added Gaussian noise with a mean of 0 and standard deviation of 0.025 to the reward probabilities. To manipulate the demands on the updating of reward value representation we applied two types of random walks with different reflecting boundaries: in the narrow probability range condition the random walks had reflecting boundaries of 0.25 and 0.75 (Daw et al., 2011). In the wide probability range condition we increased the reflecting boundaries from 0.00 to 1.00 (for examples see Figure 1A). The broader reflecting boundaries in the wide probability range condition result in more differentiable random walks for the four second-stage options. Participants performed 201 trials with the narrow random walk and 201 trials with the wide random walk in two separate sessions.

To improve subject's understanding of the task structure we designed a cover story for the task. The cover story is about a businessman who has to decide between two airplanes each of which will bring him to one of two islands (see Figure 1A). The airline is called “Surprise” and is somewhat unreliable with respect to its destinations (the transition probabilities are made explicit and are practiced by the participants). At each of the islands the businessman can trade with one of two populations of inhabitants (represented by the GoGo Figures). The productivity of the populations changes across time. The task of the businessman is to make as much money as possible by integrating information about the reward probability on the second stage and the transition structure on the first stage.

### Procedure

Participants performed two sessions, which were separated by a minimum of 1 week and a maximum of 3 weeks. In the first session participants performed a demographic questionnaire, the BIS/BAS personality questionnaire (Carver and White, 1994), Raven's progressive matrices (Raven et al., 1998) and one version of the two-stage Markov decision task (either with narrow or wide probability range condition). In the second session subjects performed an automated version of the Operation Span Task (Unsworth et al., 2005), Spot-a-Word and the Identical pictures test (Li et al., 2004), a version of the two-stage Markov decision task and an additional experimental task, data of which will be presented elsewhere. Half of the participants in each group performed the narrow probability range condition first and vice versa for the second half of the samples. Participants were informed about the nature of the transition probability structure. We also explained (and showed) to the subjects that the likelihood of getting a reward at the second stage varies over time and differs between sessions.

Prior to the task in the first session, participants completed a computerized training session, which was supervised by instructed student research assistants. In the first part of the training participants were introduced to the reward probability structure of the second (model-free) stage of the task. To familiarize participants with probabilistic reward they had to first perform 10 choices between options with a fixed reward probability of 60%. To support the understanding of probabilistic information we always referred to reward probabilities in terms absolute numbers (i.e., getting reward in 6 of 10 cases). Thereafter, participants were given 20 additional trials, in which they had to find the option with the highest reward probability (out of four choice options). After making sure that everyone found the best option we explained that the reward probabilities would change slowly across the experiment. For illustration purposes two examples of the random walks (see Figure 1) were shown in a graph.

In the next training phase participants were introduced to the transition probability structure on the first stage. That is, we informed them about the fact that there are common (characteristic) and rare (uncharacteristic) transitions and showed them a graphical picture of the transition structure (similar to Figure 1). Then participants performed 20 trials in which they practiced the transitioning from the first stage options to the second stage options (without receiving a reward). Finally, subjects played 30 trials of the experimental task (involving all stages as well as probabilistic rewards) using a different stimulus set [for similar procedures see Daw et al. (2011)]. Before the task in the second session participants performed a short practice session of 20 min. Reward was accumulated across sessions and participants were compensated according to their earnings in the task.

### Data analysis

Stay-switch behavior at the first stage was analyzed using Matlab (MATLAB, Mathworks Inc, Natick, MA) and SAS (SAS Institute Inc, Cary, NC). We defined stay-switch behavior as the probability to repeat a choice on the first stage as a function of the transition (common or rare) and the outcome (reward, no reward) on the previous trials. Mean stay probabilities were analyzed using a repeated measures ANOVA with the between subjects factors Age Group (younger, older) and WM capacity (high, low), as well as the within subjects factors probability range (narrow, wide), previous transition type (common, rare) and previous outcome (reward, no-reward). For follow-up analyses we calculated differences measures for model-free behavior [(common reward + rare reward) − (common no reward + rare no reward)] and model-based behavior [(common rewarded + rare unrewarded) − (rare rewarded + common unrewarded)] (see Figure 1B). The model-based and model-free difference values were analyzed using an ANOVA with the factors age group and range of reward probability.

### Computational model

Choice behavior was fit using a hybrid RL algorithm (Daw et al., 2011; Wunderlich et al., 2012). This algorithm assumes that choices on the first stage of the task are driven by a weighted combination of model-based RL, which accounts for the transition structure, and model-free SARSA (λ) TD learning. The weighting of model-based vs. model-free decision mechanism is determined by the free parameter omega, ω, which is held constant across trials and is constrained from 0 to 1. If ω approaches 0 behavior is model-free, which is reflected in a main effect of reward (see Figure 1B). In contrast, an omega close to 1 indicates model-based choice behavior, which is reflected in an interaction between transition structure and reward on the previous trial (see Figure 1B). Participants are assumed to select actions stochastically according to a softmax function. The choice probabilities were determined by the state-action values. For the model-fit we estimated the free parameters of the hybrid model for each probability range and subject individually via maximum likelihood. We first iterated all parameters individually by using a grid search to get a rough estimate. Subsequently, we extracted the twelve best fitting parameter combinations of both probability ranges and entered them as starting points for a precise parameter estimation, using Matlab routine fMincon.

The task consists of two stages and three states (first stage: *S*_*A*_; Second stage: *S*_*B*_, *S*_*C*_) (see Figure 1A). Each state is associated with two actions (*a*_*A*_, *a*_*B*_). At both stages (i) a state-action value function *Q*_*Si*_(*a*) is learned that maps each state action pair to its expected value. We refer to the model-based value function at the first stage as *Q*_*S*1^*MB*^_ and to the model-free value function as *Q*_*Si*^*MF*^_.

### Model-free state action values

Model-free state action values at the second stage were updated using SARSA(λ) temporal difference learning (Rummery and Niranjan, 1994). The state-action pairs were updated in each trial t according to:

$$Q_{S2^{MF}}\left(a,t+1\right)=Q_{S2^{MF}}\left(a,t\right)+\alpha_{2}(r(t)-Q_{S2^{MF}}\left(a,t\right))$$

where α_*i*_ is the learning-rate at a given stage (here stage 2) and *r*(*t*) is the received reward in that trial.

The state-action value and the reward at the second stage are then used to update the model-free values for the next choice at the *first stage* of the next trial. This updating mechanism followed the same temporal difference learning rule, with an additional parameter, λ allowing eligibility traces:

$$\begin{array}{l} Q_{S1^{MF}}\left(a,t+1\right)=Q_{S1^{MF}}\left(a,t\right)+\alpha_{1}(Q_{S2^{MF}}\left(a_{\text{chosen}},t\right)\\ \;-Q_{S1^{MF}}\left(a,t\right))+\alpha_{1}\lambda \left((r(t)-Q_{S2^{MF}}\left(a,t\right)\right) \end{array}$$

Note that eligibility traces are not assumed to carry over from trial to trial. The reason for this is the task structure that involved changing reward probabilities (the random walks) for each option across trials.

### Model-based state-action values

Model-based state-action values are computed using Bellman's equation by taking the model-free state-action values from the second stage and the transition probabilities into account.

$$\begin{array}{l} Q_{S1^{MB}}\left(a_{1}\right)=\text{HighTran}*\max\left[Q_{S2-B}^{MF}\left(a\right)\right]\\ \;+\text{LowTran}*\max\left[Q_{S2-C}^{MF}\left(a\right)\right]\\ Q_{S1^{MB}}\left(a_{2}\right)=\text{LowTran}*\max\left[Q_{S2-B}^{MF}\left(a\right)\right]\\ \;+\text{HighTran}*\max\left[Q_{S2-C}^{MF}\left(a\right)\right] \end{array}$$

In this equation “HighTran” is defined as the highest transition probability of the current condition (0.7) and ”LowTran” is defined as the lowest transition probability of that condition (0.3). Before each block participants were explicitly instructed about the nature of the transition probabilities and practiced the transitioning between states.

In the full hybrid model the *Q*_net_ state-action value was calculated as the weighted sum of model-based and model-free values:

$$Q_{S1^{\text{net}}}=\omega *Q_{S1^{MB}}\left(a\right)+\left(1-\omega \right)*Q_{S1^{MF}}\left(a\right)$$

where ω is the weighting parameter. At the second stage the *Q*_net_ state-action value is equal to the model-free state-action value (*Q*_*S*2^net^_ = *Q*_*S*2^*MF*^_).

### Softmax rule

Choice probabilities at the first stage were calculated according to a softmax rule:

$$\begin{array}{l} P_{S_{i}}\left(a_{1,t}\right)=\\ \frac{\text{exp}\left(\beta_{i}*\left[Q_{S_{1}^{\text{net}}}\left(a_{1,t}\right)+\pi *rep(a_{1})\right]\right)}{\left(\text{exp}\left(\beta_{i}*\left[Q_{S_{1}^{\text{net}}}\left(a_{1,t}\right)+\pi *rep(a_{1})\right]\right)+\text{exp}\left(\beta_{i}*\left[Q_{S^{{\text{net}}_{1}}}\left(a_{2,t}\right)+\pi *rep(a_{2})\right]\right)\right)} \end{array}$$

where β_*i*_ is the inverse softmax temperature paramter controlling the distinctiveness of the choices. We allowed both learning parameters (α_1_, α_2_) and the softmax temperature parameters (β_1_, β_2_) to differ between the two stages. The indicator function rep(a) is defined as 1 if *a* is a top-stage action and is the same as was chosen on the previous trial, zero otherwise. Taken together, the function rep(a) and the parameter π capture the degree of perseveration at the first-stage (π > 0) or the switching (π < 0) at first-stage options (Lau and Glimcher, 2005).

Choice probabilities at the second stage were calculated as follows:

$$P_{S_{i}}\left(a_{1,t}\right)=\frac{\text{exp}\left(\beta_{i}*Q_{S_{2}^{\text{net}}}\left(a_{1,t}\right)\right)}{\left(\text{exp}\left(\beta_{i}*Q_{S_{2}^{\text{net}}}\left(a_{1,t}\right)\right)\right)+\left(\text{exp}\left(\beta_{i}*Q_{S_{2}^{\text{net}}}\left(a_{2,t}\right)\right)\right)}$$

The model contained 7 free parameters (α_1_, α_2_, β_1_, β_2_, π, λ, ω). The median parameter values are shown separately for the two age groups, the two performance groups and the two probability ranges in Table 2.

**Table 2:** **Computational model parameters (Median, 25th and 75th percentile) as a function of Age group (Younger older adults) and WM Performance group (low performers, high performers) and Transition probability range (narrow, wide)**.

**Age/performance**	**Probability range**	**Parameter**	**β_1_**	**β_2_**	**α_1_**	**α_2_**	**λ**	**π**	**ω**	**−LL**
*Younger adults Low performance group*	Narrow	25th percentile	2.33	2.64	0.24	0.30	0.05	0.10	0.09	180.04
		Median	3.91	4.71	0.63	0.49	0.23	0.21	0.57	208.55
		75th percentile	8.90	5.81	0.85	0.69	0.52	0.42	0.69	231.83
	Wide	25th percentile	2.63	2.83	0.26	0.37	0.03	0.06	0.15	170.27
		Median	5.12	4.02	0.52	0.56	0.17	0.16	0.57	200.94
		75th percentile	10.37	5.21	0.86	0.73	0.62	0.33	0.79	220.00
*Younger adults High performance group*	Narrow	25th percentile	3.96	3.67	0.04	0.52	0.00	0.05	0.21	226.90
		Median	7.63	4.82	0.39	0.64	0.09	0.11	0.61	191.52
		75th percentile	10.73	6.04	0.67	0.86	0.89	0.167	0.74	171.43
	Wide	25th percentile	3.46	6.57	0.07	0.39	0.03	0.09	0.34	159.39
		Median	9.99	4.59	0.40	0.65	0.15	0.12	0.64	183.55
		75th percentile	12.39	3.78	0.58	0.85	0.36	0.24	0.78	214.80
*Older adults Low performance group*	Narrow	25th percentile	2.41	1.83	0.18	0.05	0.09	0.09	0.08	249.69
		Median	5.83	2.92	0.65	0.33	0.33	0.18	0.24	218.33
		75th percentile	9.44	5.24	0.95	0.87	0.90	0.27	0.67	188.11
	Wide	25th percentile	2.64	1.43	0.04	0.08	0.02	0.05	0.15	161.15
		Median	6.12	3.36	0.33	0.44	0.33	0.14	0.35	208.76
		75th percentile	13.54	4.91	0.82	0.88	0.83	0.29	0.49	234.82
*Older adults High performance group*	Narrow	25th percentile	2.19	1.98	0.04	0.11	0.11	0.04	0.01	170.26
		Median	3.62	2.69	0.40	0.56	0.38	0.18	0.15	202.09
		75th percentile	6.42	6.20	0.84	0.90	0.82	0.35	0.48	246.27
	Wide	25th percentile	2.52	2.10	0.09	0.06	0.08	0.04	0.01	148.89
		Median	6.32	3.75	0.62	0.32	0.49	0.18	0.11	187.34
		75th percentile	8.98	5.89	0.91	0.69	0.80	0.35	0.48	232.80

### Model fits

An ANOVA with the factors age group, performance group and probability range on the negative log-likelihoods (-LL) showed no significant age differences in the model-fits (*p* = 0.79) and no significant difference between WM capacity groups (*p* = 0.16) (see Table 2). However, we obtained a significant main effect of probability range, indicating better model fits for the wide compared to narrow probability range (*p* = 0.004, η^2^ = 0.07). Taken together, these results show comparable model fits for younger and older adults as well as for high compared to low WM capacity groups. Furthermore, as shown in Table 2 the model fits as well as the parameter estimates are comparable with those of previous studies (Daw et al., 2011; Wunderlich et al., 2012).

## Results

### Overall task performance

**Figure 2:**
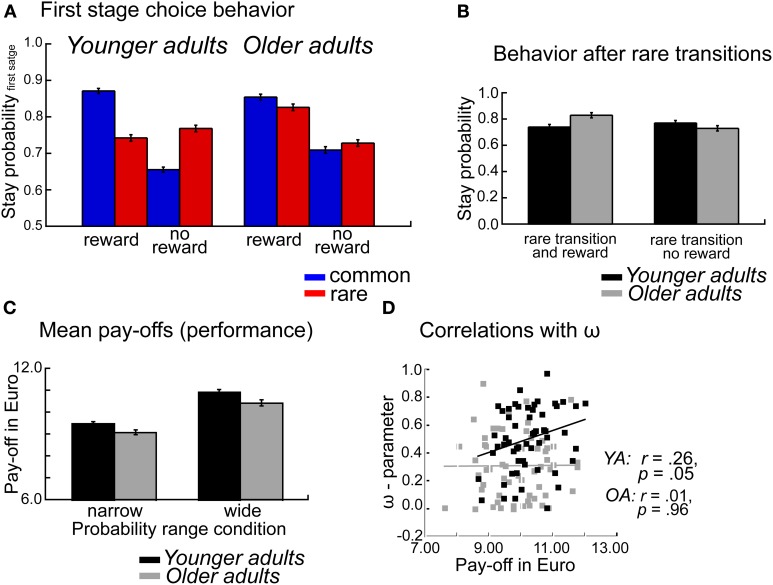
**(A)** Probability of repeating the same first stage choice as a function of the transition on the previous trials (common, rare transition) and the outcome received on the previous trial (reward, no reward). Stay probabilities are displayed separately for younger adults (left panel) and older adults (right panel), error bars reflect the standard error of the mean (s.e.m.). **(B)** Age differences in stay behavior after rare transitions as a function of age group and reward on the previous trial. **(C)** Mean pay-offs in Euro per session, displayed separately for the factors Age group and Probability range condition. **(D)** Correlations between mean pay-offs in Euro and degree of model-based choice behavior (ω-parameter).

To examine age differences in overall task performance we calculated the mean payoffs (earnings), separately for each individual and probability range condition. Mean payoffs were analyzed using an ANOVA with the between subject factors Age group, WM capacity and probability range condition. As shown in Figure 2C the analysis showed higher mean payoffs for younger compared to older adults (*p* = 0.03, η^2^ = 0.04). Furthermore, participants earned more money in the wide probability range condition compared to the narrow probability condition (*p* < 0.001, η^2^ = 0.26, see Figure 2C). No significant interaction effects were obtained (*p*'s > 19).

### Effects of age group on model-based behavior

The overall ANOVA revealed a significant interaction between age group, transition type, and outcome *F*_(1, 112)_ = 29.66, *p* < 0.001, η^2^ = 0.14. To test whether there are significant age differences in decision strategies (model-based vs. model-free) we ran an ANOVA with the factors age group, WM capacity, probability range condition and decision strategy (model-based, mb vs. model-free, mf). This analysis revealed a significant interaction between age group and decision strategy *F*_(1, 112)_ = 17.41, *p* < 0.001, η^2^ = 0.12. Separate analyses for the two decision strategies revealed significant age differences for model-based decision-making (*t* < 4.04, *p* < 0.001, η^2^ = 0.20), but not for model-free decision-making (*t* = −1.31, *p* = 0.19).

To further examine age differences in model-based behavior we performed separate analyses for the factors transition type and reward. These analyses showed a significant effect of age group only on rare rewarded trials (*t* = 2.80, *p* = 0.006, η^2^ = 0.07). To confirm that the age effect is specific to rare rewarded trials rather than rare unrewarded trials we performed a *post-hoc* contrast between the two conditions and tested for age differences. We obtained a significant interaction between age group and reward. *F*_(1, 112)_ = 17.41, *p* < 0.001, η^2^ = 0.12, indicating that older adults show enhanced stay behavior after rare transition that lead reward than younger adults, whereas age groups don't differ in their behavior after rare transitions that are followed by no reward (see Figure 2B).

Thus, age-related deficits in decision-making seem to be particularly pronounced if participants receive an unexpected reward after an uncharacteristic transition. In such a situation younger adults tend to switch to the other first stage choice option because this option is more reliably associated with the stimulus that was rewarded on the previous trial. In contrast, older adults tend to perseverate on options that were rewarded, independently of whether the reward was preceded by a common or rare transition (see Figure 2A).

### Effects of WM capacity and age differences in model-based behavior

**Figure 3:**
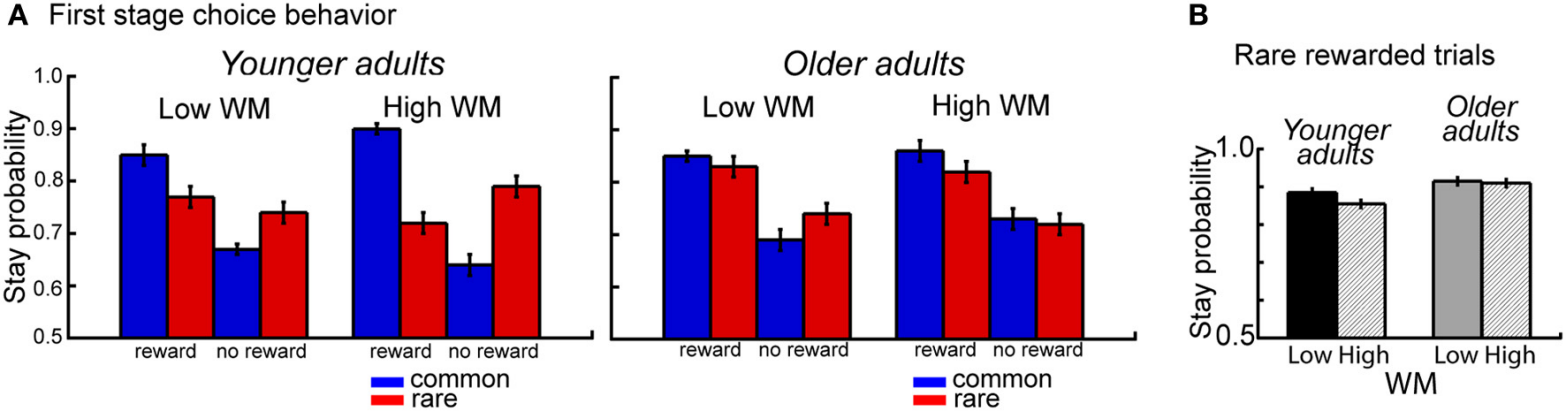
**(A)** Probability of repeating the same first stage choice as a function of the transition on the previous trials (common, rare transition) and the outcome received on the previous trial (reward, no reward). Stay probabilities are displayed separately for younger adults (left panel) and older adults (right panel) and low and high WM capacity groups. Error bars reflect the standard error of the mean (s.e.m.). **(B)** Age differences in stay behavior after rare transitions and reward on the previous trial as a function of age group and WM capacity.

The overall analysis also revealed a significant interaction between age group, WM capacity, transition type and outcome, *F*_(1,112)_ = 10.14, *p* = 0.002, η^2^ = 0.04. Separate analyses for the two age groups showed a significant interaction between WM capacity, transition type and outcome for younger adults (*p* = 0.007, η^2^ = 0.09), but not for older adults (*p* = 0.20). Analyses of the difference values showed enhanced model-based choice behavior in high WM capacity younger adults compared to low WM capacity younger adults (*t* = 2.83, *p* = 0.007, η^2^ = 0.14) but no effects of WM capacity in older adults (*t* = −1.30, *p* = 0.20) (see Figure 3A). Follow-up analyses for the factors WM capacity, transition type and reward revealed greater switching behavior after rare transitions that were followed by reward for high performing younger compared to high performing older adults (*t* = 2.59, *p* = 0.01, η^2^ = 0.11). As shown in Figure 3B, younger adults with high WM capacity show enhanced switching behavior after rare transitions that were followed by reward.

### Effects of probability range on model-based behavior

**Figure 4:**
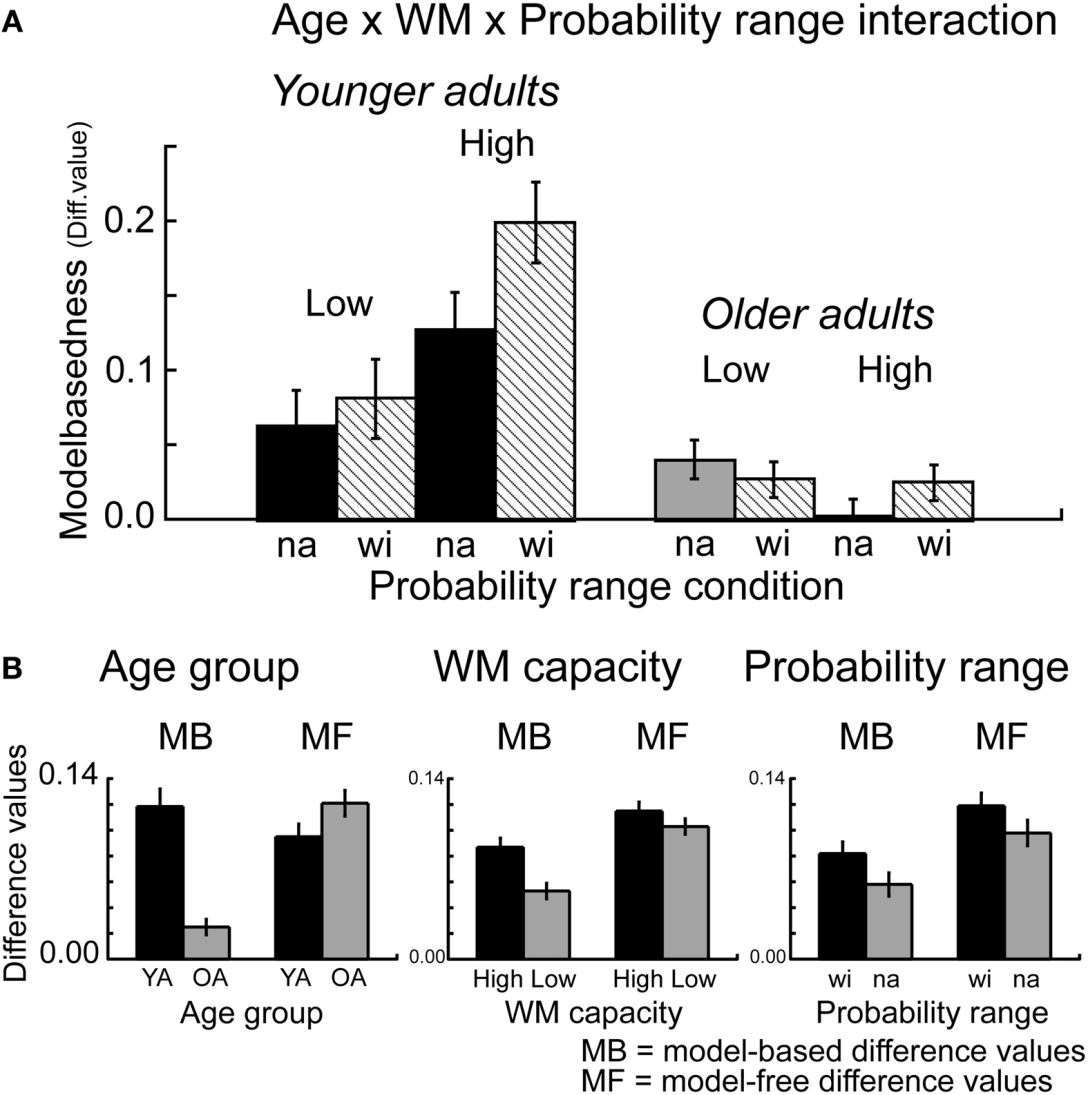
**(A)** Difference values (proportion stay trials) for model-based behavior [(common rewarded + rare unrewarded) − (rare rewarded + common unrewarded)]. Model-based differences values are shown separately for the factors Age group (younger, older adults), WM capacity (high, low capacity) and probability range (narrow, wide probability range). Error bars reflect the standard error of the mean (s.e.m.). **(B)** Model-based (MB) and model-free (MF) differences values, displayed separately for the factors Age group, WM capacity and Probability range condition. Error bars reflect the standard error of the mean (s.e.m.).

Furthermore, the overall ANOVA revealed a significant interaction between probability range, transition type and outcome *F*_(1, 112)_ = 5.16, *p* = 0.02, η^2^ = 0.05, as well as between WM capacity, probability range, transition type and outcome *F*_(1, 112)_ = 4.24, *p* = 0.04, η^2^ = 0.04. Separate analyses for the factor WM capacity showed a significant interaction between probability range, transition type and reward for high WM capacity groups (*p* < 0.006, η^2^ = 0.12) but not for low capacity groups (*p* = 0.60). Analyses of the difference values showed enhanced model-based choice behavior in the wide probability range compared to the narrow probability range for high capacity groups (*t* = 2.76, *p* = 0.008, η^2^ = 0.13) but not for low WM capacity groups (*t* = 0.18, *p* = 0.86) (see Figure 4). Separate analyses for the factors transition type and reward showed a significant main effect of probability range only for common rewarded trials (*t* = 2.97, *p* < 0.004, η^2^ = 0.08). These results suggest that the effects of probability range on model-based behavior are primarily driven by enhanced stay behavior after common transitions that were followed by reward.

### Generalized linear mixed model analysis

Given that the first stage choice proportions are binomial data (and may hence not be normally distributed) we also used a mixed logit model (mixed effects logistic regression) as implemented in the lme4 package (Bates et al., 2013) in the statistical software R (R Development Core and Team, 2010) to fit choice behavior [see also Daw et al., 2011]. The design involved the same factor structure as for the repeated measures ANOVA. The analysis revealed qualitatively similar results as the overall results from ANOVA described above. We obtained a significant interaction between age group, transition type and outcome (*p* < 0.001), reflecting greater model-based choice behavior in younger than older adults (see Figure 2 and Table 3). We also found a significant interaction between age group, WM capacity, transition type and outcome (*p* < 0.001). Separate analyses for the two age groups showed enhanced model-based behavior in high compared to low WM capacity groups in younger adults (*p* < 0.001) but not in older adults (*p* = 0.69), (see Figure 3A). Furthermore, we obtained a significant interaction between the factors probability range, transition type and outcome (*p* < 0.01). As shown in Figure 4, model-based behavior seems to be more pronounced in the wide compared to the narrow probability range condition. Taken together, the results of the mixed effects logistic regression are qualitatively consistent the results of the repeated measures ANOVA.

**Table 3:** **Results of the logistic regression analysis with the between subjects factors Age group and Working memory capacity and the within subjects factors Probability range condition (walk), transition type and reward**.

**Predictor**	**Estimate**	***p*-value**
Intercept	1.35	0.08
Walk	−0.18	0.18
Transition	0.21	0.10
Reward	0.26	0.05
Age	0.08	0.87
WM	−0.17	0.73
Walk × transition	0.06	0.67
Walk × reward	−0.06	0.67
Transition × reward	1.62	<0.001
Age × walk	0.12	0.15
Age × transition	0.00	0.96
Age × reward	0.08	0.31
WM × walk	0.11	0.20
WM × transition	−0.07	0.43
WM × reward	−0.02	0.84
Age × WM	0.09	0.77
Walk × transition × reward	−0.33	0.01
Age × walk × transition	−0.02	0.85
Age × walk × reward	0.05	0.52
Age × transition × reward	−0.78	<0.001
WM × walk × transition	−0.05	0.51
WM × walk × reward	0.06	0.47
WM × transition × reward	−0.64	<0.001
Age × WM × walk	−0.10	0.06
Age × WM × transition	−0.02	0.61
Age × WM × reward	0.00	0.99
Age × walk × transition × reward	0.12	0.16
WM × walk × transition × reward	0.12	0.15
Age × WM × walk × transition	0.02	0.67
Age × WM × walk × reward	−0.06	0.20
Age × WM × transition × reward	0.33	<0.001
Age × WM × walk × transition × reward	−0.03	0.55

### WM capacity covariance analysis

To further analyze the effects of WM capacity on age differences in model-based and model-free decision-making we performed analyses of covariance (ANCOVA) with the between-subjects factor age group, within-subjects factor probability range and the (continuous) covariate WM capacity. For model-based differences values the analysis showed a significant main effect of age group *F*_(1, 112)_ = 41.96, *p* < 0.001, η^2^ = 0.15. Importantly, this effect remained significant after controlling for WM capacity *F*_(1, 112)_ = 4.39, *p* = 0.03, η^2^ = 0.02, indicating that additional factors contributes to age differences in model-based behavior beyond the effects of WM capacity. Furthermore, we obtained a significant interaction between age group and WM capacity *F*_(1, 112)_ = 12.19, *p* < 0.001, η^2^ = 0.04. Separate analyses for the two age groups showed a significant effect of WM capacity for younger adults (*t* = 3.06, *p* = 0.002, η^2^ = 0.08), but not for older adults (*t* = 0.69, *p* = 0.87). No significant effects of age group, WM capacity or probability range were obtained for model-free differences values (*p*'s > 0.14). Taken together, these results line up with the findings of the median split analysis and show that in younger adults enhanced WM is associated with greater model-based behavior, which is not the case in older adults. Importantly, the results also show that age differences in model-based behavior remain, even after controlling for WM capacity.

### Modeling results

**Figure 5:**
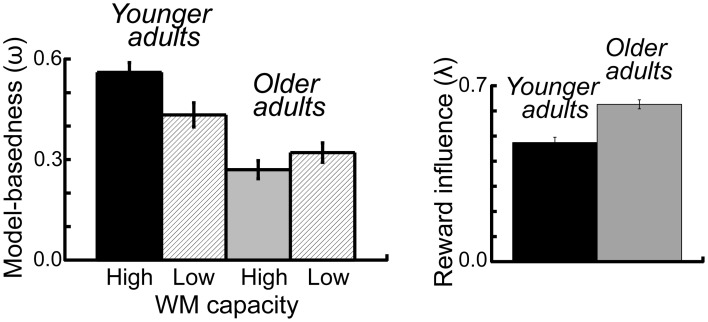
**Left panel:** Mean parameter estimates for the omega (ω-) parameter, displayed separately for the two age groups and the two WM capacity groups. The (ω-) parameter reflects the relative contribution model-based and model-free mechanisms to first stage choice behavior. **Right panel**: Mean parameter estimates for the lambda (λ-) parameter, displayed separately for the two age groups. The (λ-) parameter reflects the direct influence of reward on the previous trial on first stage choice behavior. Error bars reflect the standard error of the mean (s.e.m.).

To examine the effects of age group and WM capacity on the model parameters we applied an ANOVA with the between subjects factors age group and performance group and the within subjects factor probability range. For the ω-parameter, we found a significant main effect of age group *F*_(1, 112)_ = 20.42, *p* < 0.001, η^2^ = 0.15, As shown in Figure 5 younger adults showed a higher degree of model-based decision-making (as reflected in the ω-parameter) than older adults. Furthermore, we obtained a significantly greater λ-parameter for older compared to younger adults, *F*_(1, 112)_ = 7.72, *p* = 0.006, η^2^ = 0.06 (see Figure 5). This finding indicates that in older adults the reward received on the second stage has a greater impact on choice behavior on the first stage than this is the case in younger adults. For the inverse temperature parameter on the first stage (β_1_) we found a significant interaction between age group and WM capacity, *F*_(1, 112)_ = 5.13, *p* = 0.03, η^2^ = 0.04. Separate analyses for the two age groups showed a more differentiated choice pattern in high WM capacity compared to low WM capacity younger adults (*t* = 2.02, *p* = 0.05, η^2^ = 0.07) but no effect of WM capacity in older adults (*t* = −1.19, *p* = 0.24), (see Table 2). Finally, we obtained a significant main effect of age group on the learning rate α_1_ on the second stage *F*_(1, 112)_ = 4.2, *p* = 0.04, η^2^ = 0.04. As shown in Table 2, younger adults had a lower learning rate on the second stage than older adults, indicating that they update value representation less rapidly than older adults.

## Discussion

In this study we examined age-related and individual differences in habitual (model-free) and goal-directed (model-based) decision-making. Specifically, we were interested in three major questions: (a) Does aging affect the balance between model-based and model-free decision mechanisms? (b) Are age-related changes in decision mechanisms related to age differences in WM capacity, and (c) Can model-based behavior be supported by manipulating the distinctiveness of the reward value of the different choice options? To examine these questions, we used a two-stage Markov decision task that allows us to separate the contributions of model-free and model-based decision processes to choice behavior (Daw et al., 2011; Wunderlich et al., 2012). To support model-based behavior in this task we manipulated the range of the reward probabilities associated with the different options on the second stage (see Figure 1A). More differentiable reward probabilities on the second stage should support the ability to make deliberate, goal-directed decisions on the first stage and may hence be protective against age-related deficits in model-based decision-making. Furthermore, we acquired a WM capacity measure to investigate the impact of WM capacity on individual differences in model-based decision-making automated operation span, (Unsworth et al., 2005). Based on these WM capacity scores we separated younger and older samples into high and low WM groups.

### Age-related impairments in model-based decision-making

The analysis of the stay-switch behavior on the first stage revealed significant impairments in model-based decision-making in older adults (see Figure 1A). In contrast, no significant age differences in model-free decision-making were obtained (see Figures 1A, 4B). An analysis of age differences in the model-parameters supports these findings by showing a significant age-related reduction of the ω- parameter, which reflects the relative contribution of model-based compared to model-free decision processes to choice behavior on the first stage of the task (see Figure 5). An analysis of overall task performance showed higher mean pay-offs in younger than older adults, indicating that the more model-based strategy in younger adults is beneficial in terms of overall performance (Figure 2C). Furthermore, correlation analyses showed that in younger adults greater model-based behavior is associated with higher mean pay-offs. This is not the case in older adults (see Figure 2D). Thus, older adults who engage in a more model-based strategy do not seem to benefit from it in terms of overall performance. One interpretation of this effect might be that even though those older adults make strategic decisions on the first stage, they do not consistently choose the option with the highest expected value on the second stage. That is, overall deficits in task performance in older adults may reflect problems in the integration of model-free and model-based information.

Interestingly, age-related deficits in model-based decision-making seem to be particularly pronounced if participants receive an unexpected reward after an uncharacteristic transition and have to revise their decision strategy (see Figure 2B). In such a situation younger adults tend to switch to the other first stage option because this option is more reliably associated with the stimulus that was rewarded on the previous trial. This switching behavior can be understood in terms of a model-based exploration in which the younger adults switch to a state that may offer a greater probability of reward than the one they currently exploit. In contrast, older adults tend to perseverate on options that were rewarded, independently of whether the reward was preceded by a common or rare transition. Therefore, the current results suggest that older adults have deficits in applying their knowledge of the task structure if the reward on the previous trial reinforces stay behavior, whereas the fact that it was an uncharacteristic transition indicates the need for a shift in the response strategy on the first stage.

This interpretation is supported by two results of the modeling analysis: first, older adults show a higher λ- parameter than younger adults (see Figure 5). The λ- parameter reflects the direct influence of reward on the previous trial on stay-switch behavior on the first stage. That is, a high λ- parameter in older adults indicates that their choice behavior on the first stage is primarily influenced by the outcome on the previous trial rather than their representation of the expected value of the choice options on the previous trial. Second, we found a higher learning rate for older than younger adults on the second stage of the task. This result indicates that older adults are less consistent in their choice behavior on the second stage of the task, which may lead to deficits in building up differentiated reward value representations. Thus, our results are in line with previous findings that point to age-related impairments in the representation and updating of the expected value of choice options during RL (Eppinger et al., 2008; Eppinger and Kray, 2011; Hämmerer et al., 2011; Pietschmann et al., 2011). Furthermore, our findings line up with data from neuroimaging studies, which indicate that impairments RL in older adults are associated with age-related deficits in striatal reward prediction error signaling (Chowdury et al., 2013; Eppinger et al., 2013). However, it seems also plausible that age-related deficits in model-based decision-making are due to more complicated neuromodulatory effects in higher-order cortical areas, particularly the ventromedial and lateral prefrontal cortex. Consistent with such view, recent findings from Samanez-Larkin et al. (2012) suggest that age-related deficits in reward-based learning are, at least partially, mediated by decreased white matter integrity in fronto-striatal pathways (Samanez-Larkin et al., 2012).

Taken together, the current results suggest that age-related impairments in the updating of reward value representations may lead to deficits in goal-directed decision-making in older adults. These deficits are particularly pronounced if reward on the previous trial reinforces stay behavior, whereas the fact that it was an uncharacteristic transition indicates the need for a shift in the response strategy on the first stage. In these situations younger adults use their knowledge of the task structure to engage in strategic exploratory behavior, whereas older adults perseverate on the option they are currently exploiting.

### Effects of WM capacity and age group on model-based behavior

To examine the effects of individual differences in WM capacity on model-based behavior in the two age groups, we acquired a WM measure automated operation span, (Unsworth et al., 2005) and subdivided the younger and older adult samples into high and low WM capacity groups. We found enhanced model-based behavior for high capacity compared to low capacity younger adults, but no effect of WM capacity in older adults (see Figure 3A). Moreover, similar to the age-effects on model-based behavior, WM capacity-related differences in younger adults were most pronounced in switching behavior after rare transitions that were followed by reward (see Figure 3B). These results suggest that WM capacity is an important determinant of whether individuals engage in a model-based or model-free decision strategy. Furthermore, high WM capacity in younger adults seems to be associated with greater ability for strategic exploratory behavior. The results in younger adults are consistent with recent findings from a study that used the two stage Markov decision task in combination with a concurrent WM manipulation (Otto et al., 2013). Results of this study showed that taxing WM disrupts model-based behavior in younger adults.

What remained unclear from this study is at which decision stage the effects of WM occur. This is an interesting question, because on the one hand, WM may play a role for the representation and maintenance of the state actions values of the different options on the second stage. On the other hand, WM might also play role while trying to integrate model-free information with information about the transition structure on the first stage of the task (Gershman et al., 2013; Otto et al., 2013). The current findings show that in younger adults the effects of WM capacity are enhanced if the reward probabilities of the different options are more differentiable from each other (in the high probability range condition, see Figure 4). Hence, our findings seem more consistent with the first view, suggesting that enhanced WM capacity is associated with a greater ability to maintain model-free value representations and use them for model-based decision-making.

### Absence of WM effects on model-based behavior in older adults

**Figure 6:**
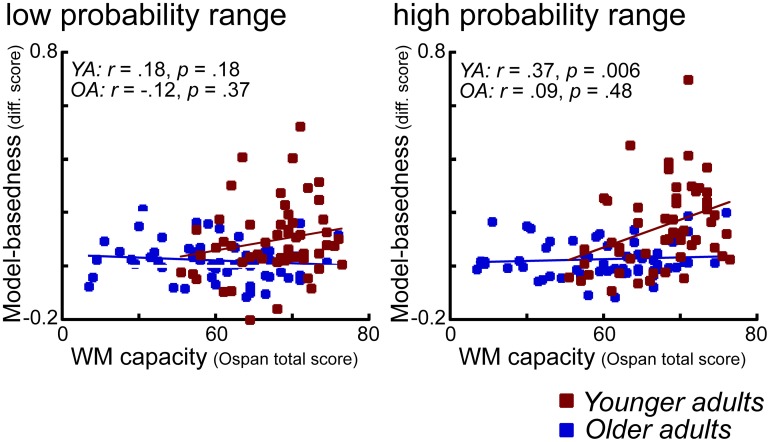
**Scatter plots for the correlations between model-based difference values (see Methods) and WM capacity (OSPAN total scores), displayed separately for the low and the high reward probability range conditions.** Younger adults are shown in red, older adults are shown in blue.

As shown in Figure 6 in older adults we found no significant correlation between WM capacity and model-based behavior. In contrast, in younger adults enhanced WM capacity is associated with a higher degree of model-based behavior, particularly in the high probability range condition. At first sight, one way to interpret these effects would be in terms of a floor effect in WM capacity in older adults. However, as also shown in Figure 6, even those older adults with high WM capacity (comparable to young high performing individuals), did not show evidence for enhanced model-based behavior. These findings suggest that factors other than WM might explain the age-related decline in model-based behavior. This interpretation is backed-up by the results of a covariance analysis which show that age differences in model-based behavior remain significant even after controlling for the effects of WM capacity. The question is what those factors might be. Consistent with the interpretation offered above, it could be argued that deficits in the updating of expected reward value might lead to these impairments. However, it could also be argued that that these impairments are due to more complex interactions between areas that represent the expected value of options and areas that are involved in implementing strategic operations, such as the lateral PFC. Results from a recent fMRI study using a three state Markov learning task suggest that age-related impairments in learning of higher order transition structures (models) are associated with a reduced recruitment of the lateral prefrontal cortex (Eppinger et al., 2012). Furthermore, results of that study indicate that model-based learning correlates positively with reasoning abilities but not with WM. Thus, these results point to the view that there is a specific deficit in older adults that relates to the learning and application of higher order associations such as sequential contingencies between events or probabilistic transition structures (such as in the current task).

Another interpretation of the absence WM effects in older adults could be that they are less willing (or able) to use an effortful decision strategy that relies on WM and rather fall back on a simpler decision strategy such as win-stay and lose-shift (Mata et al., 2010). This is somewhat supported by the modeling results, which suggest that older adults focus more on the most recent outcome than younger adults. However, given the overall performance deficits in older adults (see Figure 2C) such a strategy seems to reflect an adaptation to a behavioral impairment rather than a general difference in their approach to the task.

### Effects of probability range on model-based behavior

The analyses of the stay-switch behavior also revealed that model-based behavior is enhanced when reward probabilities on the second stage are more differentiable from each other (in the wide compared to the narrow range probability condition). Furthermore, this effect is more pronounced in high WM capacity groups compared to low WM capacity groups (see Figures 5, 6). The effects of probability range on model-based behavior are interesting for several reasons. First of all, these findings show that a manipulation that seems to primarily affect the second stage of the task can lead to a greater degree of model-based decision-making on the first stage of the task. That is, more differentiated value representations on the second stage seem to support model-based behavior on the first stage. Second, the interaction with WM capacity suggests that enhanced model-based behavior in individuals with high WM capacity may be due to a better ability to maintain and update those value representations in WM. Interestingly, a follow-up analysis of these results showed that greater model-based behavior in the wide probability range was primarily driven by enhanced stay behavior after common transitions that were followed by reward. This finding is in line with the idea that more differentiated reward probabilities on the second stage result in more consistent stay behavior on the first stage options, presumably by reducing uncertainty about the currently best option. The idea here would be that a greater distinctiveness of the value of choice options on the second stage supports the updating of those values in WM, particularly in individuals with high WM capacity. A better representation of the values of the different options on the second stage may then lead to more consistent choice behavior after common transitions that were followed by reward (i.e., in situations in which the available evidence indicates that the best thing to do is to stick to the option that has been chosen on the previous trial). Although such an interpretation seems speculative, it is consistent with theoretical ideas, suggesting that WM updating may be regulated by phasic dopaminergic prediction error signals (Braver and Cohen, 2000; Frank et al., 2001; D'Ardenne et al., 2012). According to the gating theory, it could be argued that the probability range manipulation results in more distinctive prediction error signaling and hence more reliable value representation for the different second-stage choice options. A more reliable and differentiated representation of state-action values in WM may then support the application of model-based decision strategies on the first stage of the task.

## Conclusions

Taken together, the current results show impairments in model-based decision-making in older compared to younger adults. These deficits are particularly pronounced in situations in which reward on the previous trial reinforces stay behavior, whereas the fact that it was an uncharacteristic transition indicates the need for a shift in decision strategy. In these situations younger adults engage in a strategic exploration of the task structure, whereas older adults perseverate on the option they are currently exploiting. Analyses of the model parameters showed that decision-making deficits in older adults are associated with less consistent choice patterns on the second stage and a greater direct influence of reward on the previous trial on first stage choice behavior. Thus, the current findings are consistent with the idea that age-related deficits in model-based decision-making reflect impairments in the representation and updating of expected reward value (Eppinger et al., 2011; Chowdury et al., 2013; Eppinger et al., 2013). As a consequence of those deficits, older adults rely more on the most recent outcome rather than their (impoverished) representation of the expected value of choice options on the second stage.

In addition to age-related changes in goal-directed decision-making our findings also point to substantial individual differences in model-based behavior. In younger adults high WM capacity is associated with enhanced model-based behavior. Moreover, this effect is further elevated when reward probabilities on the second stage are more differentiable from each other. The implications of these effects are two-fold: first, these findings suggest that model-based behavior is particularly prevalent in younger individuals with high WM capacity. Second, these results indicate that high WM capacity supports the ability to maintain and update (model-free) value representations and use them for strategic exploration. It could be argued that the absence of a WM effect on model-based behavior in older adults reflects a floor effect in WM capacity. However, the fact that age-related deficits in model-based behavior remain significant even after controlling for the effects of WM capacity indicates that additional factors might play a role. Based on recent fMRI findings (Eppinger et al., 2012) we argue that an under-recruitment of the lateral PFC during the integration of expected reward value into model-based decisions might be one possible explanation for these effects.

## Authors contribution

Ben Eppinger, Maik Walter, Shu-Chen Li, Hauke R. Heekeren designed the study. Ben Eppinger, Maik Walter, acquired and analyzed the data. Ben Eppinger, Maik Walter, Shu-Chen Li; Hauke R. Heekeren wrote the manuscript.

### Conflict of interest statement

The authors declare that the research was conducted in the absence of any commercial or financial relationships that could be construed as a potential conflict of interest.

## References

[B1] Baddeley A. D. Emslie H. Nimmo-Smith I. (eds.). (1992). The Speed and Capacity of Language Processing (SCOLP) Test. Bury St. Edmunds, Suffolk, England: Thames Valley Test Company

[B2] Balleine B. W. O'Doherty J. P. (2010). Human and rodent homologies in action control: cortico-striatal determinants of goal-directed and habitual action. Neuropsychopharmacology 35, 48–69 10.1038/npp.2009.131 19776734 PMC3055420

[B3] Bates D. Maechler M. Bolker B. (2013). lme4: linear mized-effects models using S4 classes. R version 3.0.2.

[B4] Braver T. S. Cohen J. D. (2000). On the control of control: the role of dopamine in regulating prefrontal function and working memory, in Attention and Performance XVIII, eds. Monsell S. Driver J. (Cambridge, MA: MIT Press), 713–737

[B5] Carver C. S. White T. L. (1994). Behavioral inhibition, behavioral activation, and affective responses to impending reward and punishment: the BIS/BAS scales. J. Pers. Soc. Psychol. 67, 319–333 10.1037/0022-3514.67.2.319

[B6] Chowdury R. Guitart-Masip M. Lambert C. Dayan P. Huys Q. Duezel E. (2013). Dopamine restores reward prediction errors in old age. Nat. Neurosci. 16, 648–653 10.1038/nn.3364 23525044 PMC3672991

[B7] D'Ardenne K. Eshel N. Luka J. Lenartowicz A. Nystrom L. E. Cohen J. D. (2012). Role of prefrontal cortex and the midbrain dopamine system in working memory updating. Proc. Natl. Acad. Sci. U.S.A.109, 19900–19909 10.1073/pnas.1116727109 23086162 PMC3523834

[B8] D'Ardenne K. Mcclure S. M. Nystrom L. Cohen J. D. (2008). BOLD Responses reflecting dopaminergic signals in the human ventral tegmental area. Science 319, 1264–1267 10.1126/science.1150605 18309087

[B9] Daw N. D. Gershman S. J. Seymour B. Dayan P. Dolan R. J. (2011). Model-based influences on humans' choices and striatal prediction errors. Neuron 69, 1204–1215 10.1016/j.neuron.2011.02.027 21435563 PMC3077926

[B10] Daw N. D. Niv Y. Dayan P. (2005). Human and rodent homologies in action control: cortico-striatal determinants of goal-directed and habitual action. Nat. Neurosci. 8, 1704–1711 10.1038/nn1560 16286932

[B11] Doll B. B. Simon D. A. Daw N. D. (2012). The ubiquity of model-based reinforcement learning. Curr. Opin. Neurobiol. 22, 1075–1081 10.1016/j.conb.2012.08.003 22959354 PMC3513648

[B12] Duncan J. Schramm M. Thompson R. Dumontheil I. (2012). Task rules, working memory and fluid intelligence. Psychon. Bull. Rev. 19, 864–870 10.3758/s13423-012-0225-y 22806448 PMC3456922

[B13] Ekstrom R. B. French J. W. Harman H. H. (1976). Kit of Factor-Referenced Cognitive Tests. Princeton, NJ: E.T. Service

[B14] Eppinger B. Haemmerer D. Li S.-C. (2011). Neuromodulation of reward-based learning and decision making in human aging. Ann. N.Y. Acad. Sci. 1235, 1–17 10.1111/j.1749-6632.2011.06230.x 22023564 PMC3779838

[B15] Eppinger B. Heekeren H. R. Li S.-C. (2012). When two birds in the bush are better than one in the hand: age-related impairments in learning to predict future rewards, in Annual Meeting of the Society for Neuroscience, (New Orleans, LA).

[B16] Eppinger B. Kray J. (2011). To choose or to avoid: age differences in learning form positive and negative feedback. J. Cogn. Neurosci. 23, 41–52 10.1162/jocn.2009.21364 19925176

[B17] Eppinger B. Kray J. Mock B. Mecklinger A. (2008). Better or worse than expected? Aging, Learning, and the ERN. Neuropsychologia 46, 521–539 10.1016/j.neuropsychologia.2007.09.001 17936313

[B18] Eppinger B. Schuck N. W. Nystrom L. E. Cohen J. D. (2013). Reduced striatal responses to reward prediction errors in older compared to younger adults. J. Neurosci. 33, 9905–9912 10.1523/JNEUROSCI.2942-12.2013 23761885 PMC3682384

[B19] Frank M. J. Kong L. (2008). Learning to avoid in older age. Psychol. Aging 23, 392–398 10.1037/0882-7974.23.2.392 18573012

[B20] Frank M. J. Loughry B. O'Reilly R. C. (2001). Interactions between frontal cortex and basal ganglia in working memory: a computational model. Cogn. Affect. Behav. Neurosci. 1, 137–160 10.3758/CABN.1.2.137 12467110

[B21] Gershman S. J. Markman A. B. Otto A. R. (2013). Retrospective revaluation in sequential decision making: a tale of two systems. J. Exp. Psychol. Gen. [Epub ahead of print]. 10.1037/a0030844 23230992

[B22] Gläscher J. Daw N. D. Dayan P. O'Doherty J. P. (2010). States versus rewards: dissociable neural prediction error signals underlying model-based and model-free reinforcement learning. Neuron 66, 585–595 10.1016/j.neuron.2010.04.016 20510862 PMC2895323

[B23] Hämmerer D. Eppinger B. (2012). Dopaminergic and prefrontal contributions to reward-based learning and outcome monitoring during child development and aging. Dev. Psychol. 48, 862–874 10.1037/a0027342 22390655

[B24] Hämmerer D. Li S.-C. Mueller V. Lindenberger U. (2011). Lifespan differences in electrophysiological correlates of monitoring gains and losses during probabilistic reinforcement learning. J. Cogn. Neurosci. 23, 579–592 10.1162/jocn.2010.21475 20377358

[B25] Kahneman D. (2011). Thinking, Fast and Slow. New York. NY: Farrar, Strauss, Giroux

[B26] Lau B. Glimcher P. W. (2005). Dynamic response-by-response models of matching behavior in Rhesus monkeys. J. Exp. Anal. Behav. 84, 555–579 10.1901/jeab.2005.110-04 16596980 PMC1389781

[B27] Li S.-C. Lindenberger U. Hommel B. Aschersleben G. Prinz W. Baltes P. B. (2004). Transformations in the couplings among intellectual abilities and constituent cognitive processes across the lifespan. Psychol. Sci. 15, 155–163 10.1111/j.0956-7976.2004.01503003.x 15016286

[B28] Mata R. Von Helversen B. Rieskamp J. (2010). Learning to choose: cognitive aging and strategy selection learning in decision making. Psychol. Aging 25, 299–309 10.1037/a0018923 20545415

[B29] Miller E. K. Cohen J. D. (2001). An integrative theory of prefrontal cortex function. Annu. Rev. Neurosci. 24, 167–202 10.1146/annurev.neuro.24.1.167 11283309

[B30] Montague P. R. Hyman S. E. Cohen J. D. (2004). Computational roles for dopamine in behavioral control. Nature 431, 760–767 10.1038/nature03015 15483596

[B31] Nieuwenhuis S. Ridderinkhof K. R. Talsma D. Coles M. G. H. Holroyd C. B. Kok A. (2002). A computational account of altered error processing in older age: dopamine and the error-related negativity. Cogn. Affect. Behav. Neurosci. 2, 19–36 10.3758/CABN.2.1.19 12452582

[B32] Niv Y. Edlund J. A. Dayan P. O'Doherty J. P. (2012). Neural prediction errors reveal a risk-sensitive reinforcement-learning process in the human brain. J. Neurosci. 32, 551–562 10.1523/JNEUROSCI.5498-10.2012 22238090 PMC6621075

[B33] Niv Y. Schoenbaum G. (2008). Dialogues on prediction errors. Trends Cogn. Sci. 12, 265–272 10.1016/j.tics.2008.03.006 18567531

[B34] Otto A. R. Gershman S. J. Markman A. B. Daw N. D. (2013). The curse of planning: dissecting multiple reinforcement learning systems by taxing the central executive. Psychol. Sci. 24, 751–761 10.1177/0956797612463080 23558545 PMC3843765

[B35] Pietschmann M. Endrass T. Czerwon B. Kathmann N. (2011). Aging, probabilistic learning and performance monitoring. Biol. Psychol. 86, 74–82 10.1016/j.biopsycho.2010.10.009 21056080

[B36] Raven J. C. Raven J. E. Court J. H. (eds.). (1998). Progressive Matrices. Oxford: Oxford Psychologists Press

[B37] R Development Core and Team (2010). R: A Language and Environment for Statistical Computing. Vienna: R.F.F.S. Computing

[B38] Rummery G. Niranjan M. (1994). On-line Q-learning Using Connectionist Systems. Cambridge: Cambridge University

[B39] Salthouse T. A. (1994). The aging of working memory. Neuropsychology 8, 535–543 10.1037/0894-4105.8.4.53516158065

[B40] Salthouse T. A. Mitchell D. R. D. Skovronek E. Babcock R. L. (1989). Effects of adult age and working memory on reasoning and spatial abilities. J. Exp. Psychol. Learn. Mem. Cogn. 15, 507–516 10.1037/0278-7393.15.3.507 2524548

[B41a] Samanez-Larkin G. R. Kuhnen C. M. Yoo D. J. Knutson B. (2010). Variability in nucleus accumbens activity mediates age-related suboptimal financial risk taking. J. Neurosci. 27, 1426–1434 20107069 10.1523/JNEUROSCI.4902-09.2010 PMC2821055

[B41] Samanez-Larkin G. R. Levens S. M. Perry L. M. Dougherty R. F. Knutson B. (2012). Frontostriatal white matter integrity mediates adult age differences in probabilistic reward learning. J. Neurosci. 32, 5333–5337 10.1523/JNEUROSCI.5756-11.2012 22496578 PMC3744863

[B42] Schultz W. Dayan P. Montague P. R. (1997). A neural substrate of predicition and reward. Science 275, 1593–1599 10.1126/science.275.5306.1593 9054347

[B43] Sutton R. S. Barto A. G. (1998). Reinforcement Learning: An Introduction (Adaptive Computation and Machine Learning). Cambridge, MA: MIT Press

[B44] Thorndike E. L. (1911). Animal Intelligence. New York, NY: Macmillan

[B45] Tolman E. C. (1948). Cognitive maps in rats and men. Psychol. Rev. 55, 189–208 10.1037/h0061626 18870876

[B46] Turner M. L. Engle R. W. (1989). Is working memory capacity task dependent. J. Mem. Lang. 28, 127–154 10.1016/0749-596X(89)90040-5

[B47] Unsworth N. Heitz R. P. Dschrock J. C. Engle R. W. (2005). An automated version of the operation span task. Behav. Res. Methods 37, 498–505 10.3758/BF03192720 16405146

[B48] Worthy D. A. Gorlick M. A. Pacheco J. L. Schnyer D. M. Maddox W. T. (2011). With age come wisdom: decision making in younger and older adults. Psychol. Sci. 22, 1375–1380 10.1177/0956797611420301 21960248 PMC3212636

[B49] Worthy D. A. Maddox W. T. (2012). Age-based differences in strategy use in choice tasks. Front. Neurosci. 5:145 10.3389/fnins.2011.00145 22232573 PMC3252562

[B50] Wunderlich K. Smittenaar P. Dolan R. J. (2012). Dopamine enhances model-based over model-free behavior. Neuron 75, 418–424 10.1016/j.neuron.2012.03.042 22884326 PMC3417237

